# Stereoscopic UWB Yagi–Uda Antenna with Stable Gain by Metamaterial for Vehicular 5G Communication

**DOI:** 10.3390/s23094534

**Published:** 2023-05-06

**Authors:** Yuanxu Fu, Tao Shen, Jiangling Dou, Zhe Chen

**Affiliations:** Faculty of Information Engineering and Automation, Kunming University of Science and Technology, Kunming 650032, Chinajianglingdou@kust.edu.cn (J.D.); chenzhe@kust.edu.cn (Z.C.)

**Keywords:** NZIM, ME structure, flat gain, quality factor Q

## Abstract

In this paper, a stereoscopic ultra-wideband (UWB) Yagi–Uda (SUY) antenna with stable gain by near-zero-index metamaterial (NZIM) has been proposed for vehicular 5G communication. The proposed antenna consists of magneto-electric (ME) dipole structure and coaxial feed patch antenna. The combination of patch antenna and ME structure allows the proposed antenna can work as a Yagi–Uda antenna, which enhances its gain and bandwidth. NZIM removes a pair of C-notches on the surface of the ME structure to make it absorb energy, which results in two radiation nulls on both sides of the gain passband. At the same time, the bandwidth can be enhanced effectively. In order to further improve the stable gain, impedance matching is achieved by removing the patch diagonally; thus, it is able to tune the antenna gain of the suppression boundary and open the possibility to reach the most important characteristic: a very stable gain in a wide frequency range. The SUY antenna is fabricated and measured, which has a measured −10 dBi impedance bandwidth of approximately 40% (3.5–5.5 GHz). Within it, the peak gain of the antenna reaches 8.5 dBi, and the flat in-band gain has a ripple lower than 0.5 dBi.

## 1. Introduction

The 5G is the current generation of mobile communications expected to provide higher data rates and high-speed connectivity for multimedia applications [1,2,3,4,5]. In addition to mobile communication, there are more usages of the 5G communication systems such as the vehicle-to-everything (V2X) technique [6,7]. On one hand, the system integration of the terrestrial and satellite communication [8,9] can provide global seamless services. It is the development vision of the next generation wireless network [10,11]. On the other hand, the 5G communication system is convenient for communication between vehicles and base stations [12,13]. In order to reduce the interference between communication systems, an antenna with high gain and good direction is urgently needed [14,15]. Among them, the Yagi antenna is particularly prominent on account of its advantages [16], such as its light weight and simple structure [17,18,19,20]. However, it usually has a narrow operating band which cannot meet the multi-frequency operational requirements of wireless communication. Accordingly, many efforts have been made to enlarge the impedance bandwidth. In [21,22], impedance matching is adopted to improve the bandwidth of the antenna. In [23,24], this operational mode adopts a ground slot to solve the bandwidth problem of the bandwidth of antennas. In [25], bandwidth enhancement adopts a semielliptical monopole as a driven element, a half bow-tie-shaped truncated ground reflector, and elliptical director elements. The above structure can not enhance the gain while improving the bandwidth.

In this paper, the magneto-electric dipole (ME) structure is used to improve the bandwidth of the antenna by impedance matching and to enhance the antenna gain by electromagnetic superposition. In order to further improve the gain of the Yagi antenna, metamaterials are proposed below to improve the Yagi antenna gain. In [26], enhanced and stable gain are achieved by a wideband metematerial-loaded loop. In [20], to improve the in-band gain and enhance the stable gain capability, a new coplanar NZIM with wideband NZI characteristic and dual transmission notches in the lower and upper stopbands of the preliminary gain passband is introduced to the Yagi antenna. In [27],the four-step slotline along the center of the antenna is used to improve the impedance matching, and the metasurface is used to enhance the gain. The above method can improve the stable gain, but the bandwidth is narrow. The narrow bandwidth causes frequency shift, efficiency, and the voltage of the antenna receiving drop, which affects the communication quality.

In this paper, we propose the SUY antenna which has ultra-wideband and stable gain for the RF energy harvesting to obtain a stable output voltage and conversion efficiency. The following three steps are used to achieve the characteristics of the ultra-wideband and stable gain: First, the ME structure is used to achieve the reinforcement of broadband and gain. Second, a pair of C-notches is cut out of the surface of the ME structure to make a near-zero-index metamaterial (NZIM). This metamaterial structure not only produces out-of-band radiation suppression by energy absorption but also increases the bandwidth of the antenna. Finally, in order to further improve the stable gain, the director and driven element achieve impedance matching for enhancement of the gain of the suppression boundary. To show the advantages of the SUY antenna stable gain, as shown in Table 1, the advantages of the SUY antenna are stable and high gain, while the disadvantage is that the three-dimensional dimension is larger. Compared with wideband Yagi antenna, it has high gain characteristics, and compared with stable gain antenna, it has wide bandwidth and high gain. We use △G to signify the difference value of gain and BW to signify bandwidth. △G/BW is defined as the standard of the stable gain of the antenna [27].

## 2. Configuration

**Figure 1:**
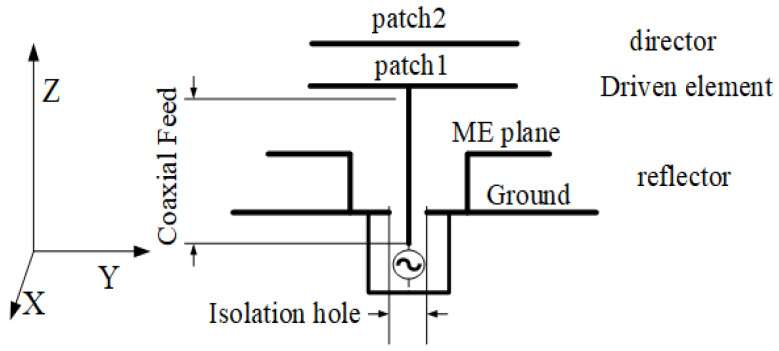
Design configuration of stereoscopic UWB Yagi–Uda.

**Figure 2:**
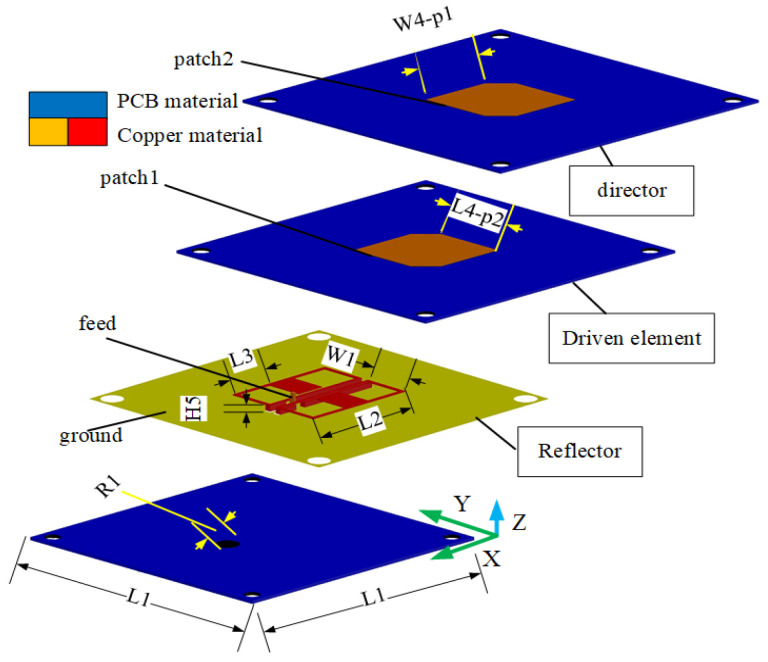
Processing drawing of the vertical plane of the stereoscopic UWB Yagi–Uda antenna.

Figure 1 shows the design configuration of the stereoscopic UWB Yagi–Uda antenna (length: 60 mm; width: 60 mm; height: 8.5 mm). The antenna consists of four parts: the driven element, director layer, ME structure, and coaxial feed. The ME structure is the reflection layer. Patch 1 and patch 2 are the driven element and director of the SUY antenna, respectively. They are the same size by impedance optimization. The two substrates are made of glass-fiber epoxy resin copper-clad laminate (FR4-epoxy). The permittivity of these substrates is 4.4, with a tangential loss of 0.0027 and a thickness of 1.5 mm. As shown in Figure 2, the ME structure is made of copper material. An F4B Rogers PCB was used for the ground substrate, which has a dielectric permittivity of 2.65, tangential loss of 0.001, and thickness of 2 mm. Table 2 shows the specific component dimension of the SUY antenna. As can be seen from Figure 2, its structure is relatively complex, but the raw materials are economical and popular. So this antenna is used in car wireless networks.

## 3. Antenna Design and Analysis

**Figure 3:**
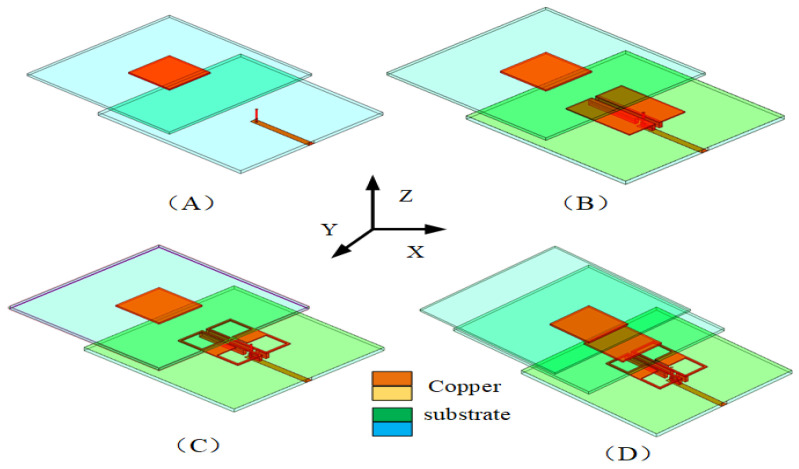
A-type (**A**), B-type (**B**), C-type (**C**), and D-type (**D**) antenna structures.

**Figure 4:**
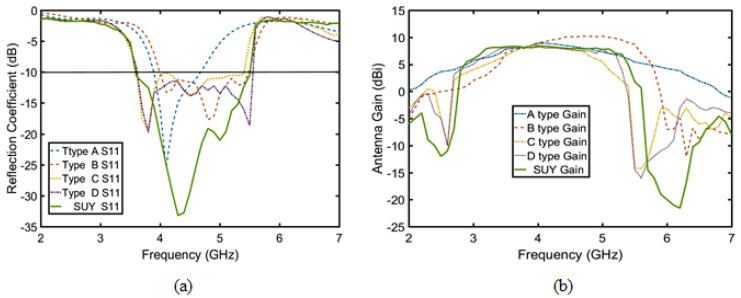
Four-antenna types and the SUY antenna: (**a**) reflection coefficient and (**b**) gain.

To analyze the SUY antenna more clearly, the SUY antenna is divided into the A-type antenna, B-type antenna, C-type antenna, and D-type antenna in Figure 3. In order to further understand the four antennas, as shown in Figure 4, the gain and reflection coefficient reflect the characteristics of each antenna. The specific characteristics are as follows: The A-type antenna is an original patch antenna with coaxial feed. The B-type antenna adopts ME structure to realize the double increase of bandwidth and gain, which appears as a zero radiation point due to current convection.

The C-type antenna realizes the filtering of out of band suppression by NZIM, which also increases the antenna bandwidth. The D-type antenna increases and flattens the gain of the upper suppression boundary at 4.9–5.5 GHz by adding director layer. The SUY antenna achieves stable the gain of lower suppression boundary from 3.5 to 4 GHz by cutting out the diagonal of patch 1 and patch 2. The four kinds of antennas are analyzed in the following sections.

### 3.1. ME Characteristic Analysis

**Figure 5:**
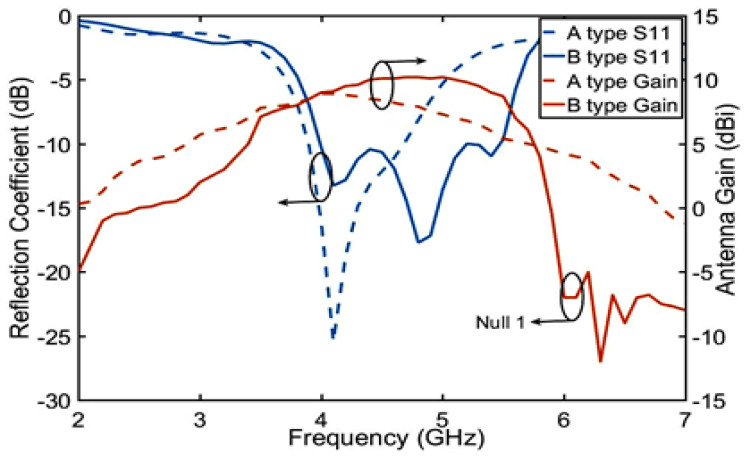
Gain and reflection coefficient simulation of A-type and B-type antennas.

The ME structure has two characteristics: one is to improve the antenna gain by the electromagnetic superposition principle and the other is its impedance matching to improve the bandwidth as shown in Equations (1) and (2). As shown in Figure 5, the B-type antenna is made by adding the ME structure to the A-type antenna, which has increased bandwidth and gain simultaneously. The gain of the B-type antenna exhibits upper stopband suppression.

**Figure 6:**
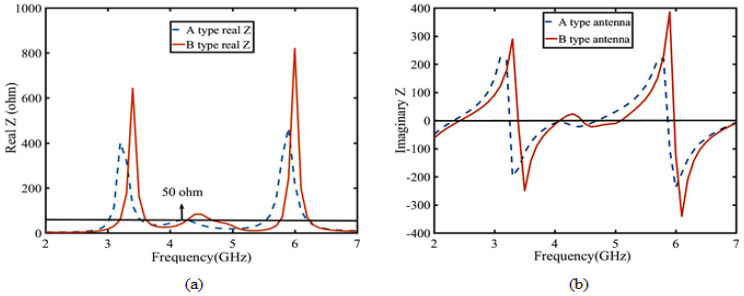
Impedance simulation of A-type and B-type antennas: (**a**) real Z and (**b**) imaginary Z.

**Figure 7:**
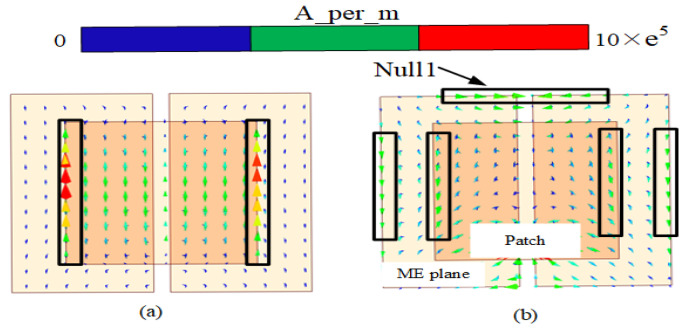
Current distribution at (**a**) 3.4 GHz and (**b**) 4.7 GHz.

**Figure 8:**
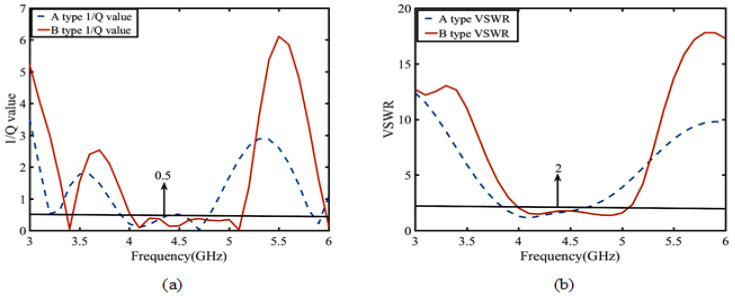
Simulation of the 1/Q value and VSWR parameter between A-type and B-type antennas: (**a**) 1/Q value and (**b**) VSWR parameter.

First, the reason for the electromagnetic superposition at 4.7 GHz is illustrated to explain that the gain of the B-type antenna can reach more than 9 dBi. As shown in Figure 6, according to the Simth circle diagram, impedance matching is best when the real part of impedance is 50 ohms and the imaginary part is 0. When the imaginary part is capacitive and the real part is greater than 50 ohms, the reflected induced current is in the same direction as the induced current of the driven element. Their phase difference is 0 degrees, which leads to high gain by electromagnetic superposition. In contrast, when the imaginary part is inductive and real part is 50 ohms smaller, the induced current in the reflection layer is opposite to that in the driven element layer with a phase difference of 180 degrees. Only the driven element current exists, so the gain at 3–4 GHz is not high gain. To demonstrate this phenomenon, the current distributions of 3.4 GHz and 4.7 GHz are shown in Figure 7. Secondly, the bandwidth of the antenna in Equation (1) is related to VSWR and 1/Q. Figure 8 shows the calculation result of 1/Q and VSWR. From the above results, it is concluded that the bandwidth of the B-type antenna is determined by Q. The change of Q value mainly depends on the impedance optimization in ME structures in Equation (2). Finally, as shown in Figure 7b, because the opposite current direction and equal current density produce zero radiation (Null 1) at 6 GHz. However, its filtering selectivity is not adequate, especially in the lower stopband suppression. We apply dual-C-notch NZIM to improve lower suppression selectivity.

(1) $$\mathrm{BW}=\frac{s-1}{\sqrt{s}Q}$$

BW is the bandwidth, s is the voltage standing wave ratio (VSWR), and Q is the quality factors [28].

(2) $$\frac{1}{Q}=\frac{Z_{\mathrm{im}}}{Z_{\mathrm{real}}}$$

$Z_{\mathrm{im}}$ is the imaginary part of the impedance, and $Z_{\mathrm{real}}$ is the real part of the impedance [29].

### 3.2. Design of the Dual-C-Notch NZIM

In this section, the dual-C-notch NZIM structure is designed to achieve two targets: one is to improve the out-of-band suppression by introducing the radiation nulls of the lower and upper gain stopbands by energy absorption and other is to enhance the bandwidth by impedance matching. This design idea of dual-C-notch NZIM is derived from absorptive-branch-loaded structure and dual-notch NZIM structure [30,31]. For convenience of expression, the dual-C-notch NZIM structure is defined as a C-type antenna (this C-type antenna is cut into dual-C-notch on the surface of the ME structure of the B-type antenna.). The dual-C-notch NZIM structure enhances the bandwidth and improves the out-of-band suppression by absorption.

**Figure 9:**
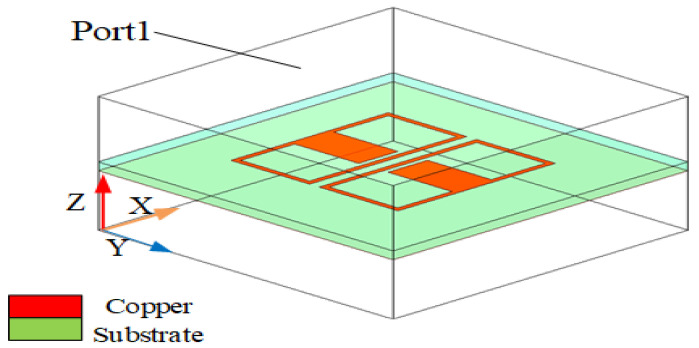
NZIM simulation: electromagnetic field boundaries.

**Figure 10:**
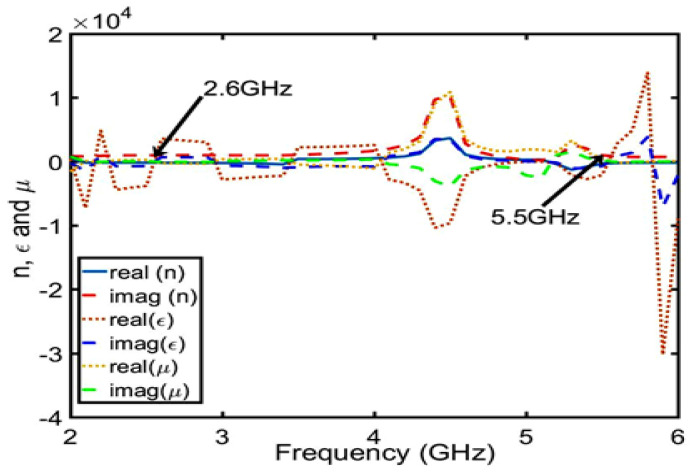
Real part and imaginary part of the permittivity (ϵ), the magnetic permeability (μ), and the refractive index (n).

**Figure 11:**
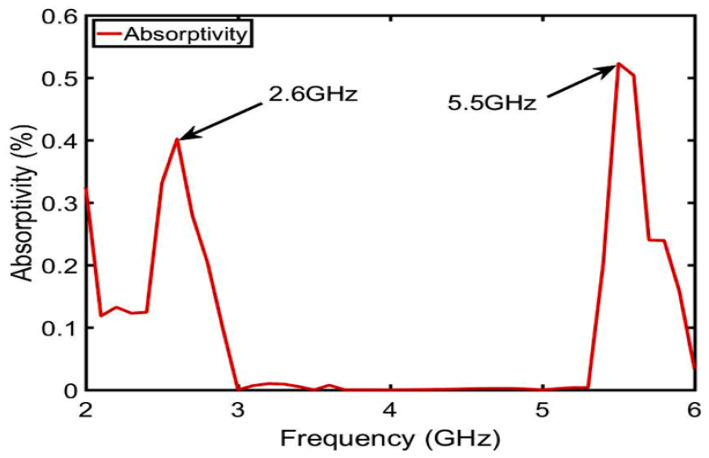
Absorption simulation.

**Figure 12:**
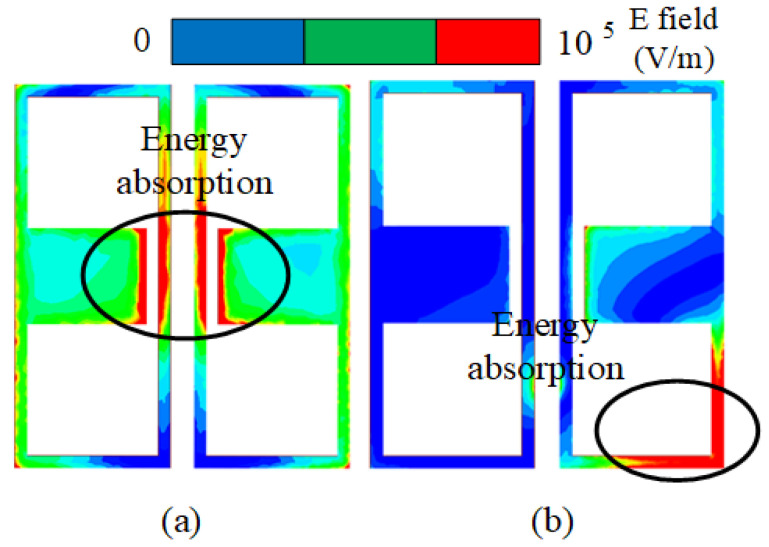
E field simulation at (**a**) 2.6 GHz and (**b**) 5.5GHz.

**Figure 13:**
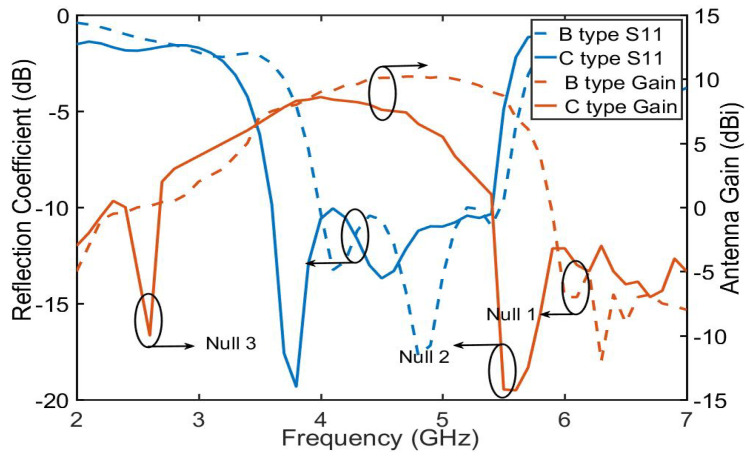
Simulation of gain and reflection coefficient of the B-type and C-type antennas.

The absorption function of NZIM is verified from two aspects: first, it is proved that the dual-C-notch belongs to NZIM at 2.6 GHz and 5.5 GHz; second, it has absorption function at 2.6 GHz and 5.5 GHz by electric field simulation. First, as shown in Figure 9, the dual-C-notch structure with periodic boundary condition is modeled to evaluate the scatting performance. Periodic electrical boundaries (PEC) are assigned along the Y-axis direction and periodic magnetic boundaries (PMC) are excited perpendicularly to the dielectric substrate. The input/output port of waveports is set along the Z-axis direction. The real and imaginary parts of the permittivity (ϵ), the magnetic permeability (μ), and the refractive index (n) by Formula (1) and the calculation method of the equivalent medium theoretical model are shown in Figure 10. It can be seen that the dual-C-notch structure conforms to the NZIM theorem because almost all parameters are near zero in the figure at 2.6 GHz and 5.5 GHz. Second, As shown in Figure 11, the absorptivity values of 2.6 GHz and 5.5 GHz frequencies are 40% and 60% respectively, and such energy absorption results in radiation attenuation. Figure 12 shows that the 2.6 GHz energy absorption position is an intermediate coupled with the dual-C-notch, while the 5.5 GHz energy absorption position is at one end of the direction loop of the dual-C-notch. The above proves the existence of zero radiation (null2 and null3), as shown in Figure 13. At the same time, the bandwidth of the C-type antenna can be shown to be increased.

(3) $$n=\pm \frac{1}{\mathrm{kd}}\left[\cos^{-1}\left(\frac{1-S_{11}^{2}+S_{21}^{2}}{2S_{21}}\right)\right]$$

where $S_{11}$ and $S_{21}$ are reflection and transmission coefficients. The n is the refractive index, k is wave number, d is the length of the unit cell [32].

### 3.3. Enhancement Suppression Boundary of Gain

In order to further improve the stable gain, the gain of the suppression boundary is enhanced to achieve two targets: one is to enhance the gain of upper suppression boundary by addition of the director layer from 4.9 to 5.5 GHz and the other is to improve the gain of lower suppression boundary by the diagonal cut of the patch from 3.5 to 4 GHz.

**Figure 14:**
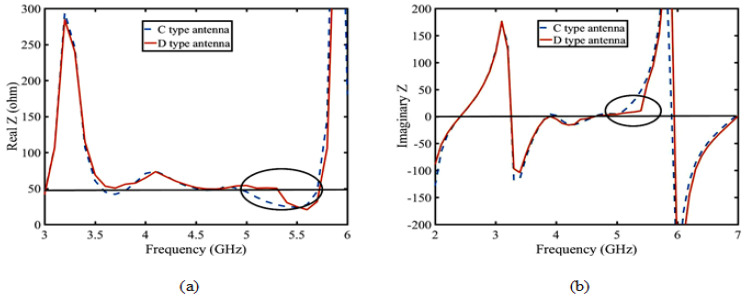
Impedance of the C-type and D-type antennas: (**a**) real Z; (**b**) imaginary Z.

**Figure 15:**
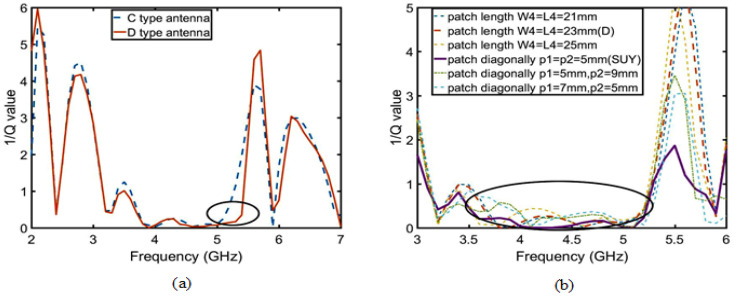
Analysis of the 1/Q value of flat gain. (**a**) The Q value of the D-type antenna is more stable than that of the C-type antenna. (**b**) The Q value of patch 1 and patch 2 diagonal cuts is more stable than that their variation in length.

Firstly, according to the Yagi antenna principle, the director layer is added to the C-type antenna to improve antenna gain. The antenna is defined as a D-type antenna. As shown in Figure 14, after adding the director layer, the real part of the D-type antenna is close to 50 ohms, and the imaginary part is close to 0 in the range of 4.9–5.5 GHz. According to the Smith circle diagram, the gain is improved by the optimal matching. According to Equations (2) and (4), the gain increases with the increase of the quality factor (Q) after impedance matching in Figure 15a. So, the D-type antenna achieves enhancement gain of the upper suppression boundary by adding director to realize impedance matching.

**Figure 16:**
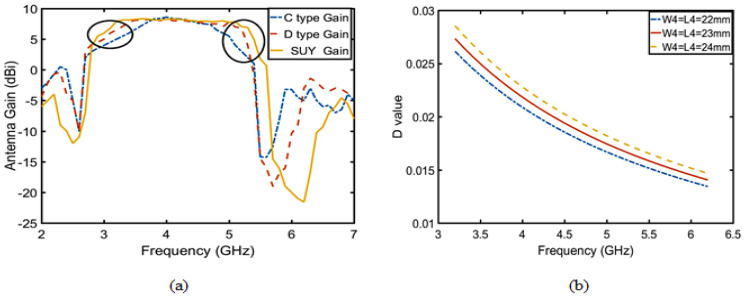
Flat gain of the SUY antenna is analyzed according to Equation (6). (**a**) Gain of the C-type, D-type, and SUY antenna and (**b**) relation between the radiation direction (D) and frequency.

**Figure 17:**
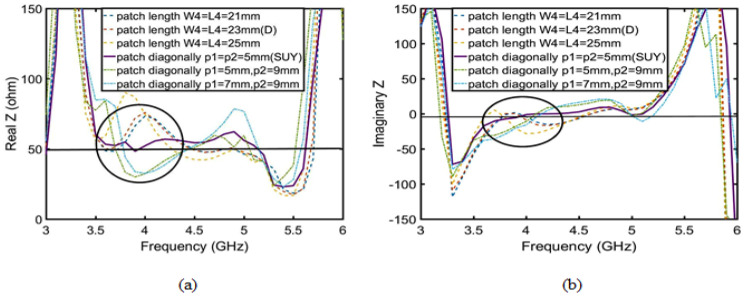
Contrast between the length variation impedance of patch 1 and patch 2 and their diagonal excision impedance: (**a**) real Z; (**b**) imaginary Z.

Secondly, there are three ways to improve the gain of upper suppression boundary: one is to add another director layer, another is to change the length of patch 1 and 2, and the last is to change the shape of patch 1 and 2. The first method reduces the overall gain of the antenna, so it is not used. The second and third methods are verified in terms of which method is suitable to improve the gain of upper suppression boundary by the quality factor (Q). According to Equations (4)–(7), where radiation loss ($Q_{r}$) is constant, only direction coefficient (D) is related to length of patch 1 and 2, but patch 1 and 2 must be square. As shown in Figure 16b, the direction coefficient (D) is less than or equal to 0.03. This means that increasing the patch length will not change the antenna gain, and only changing its shape can improve the antenna gain. The method of diagonal cut of the patch 1 and 2 increase the gain of lower suppression boundary. As shown in Figure 17, when p1 = p2 = 5 mm, impedance matching reaches the best point. Finally, the Q reaches its maximum value in Figure 15b. The enhanced suppression boundary gain is to achieve the peak gain reaching 8.5 dBi, and the flat in-band gain has a ripple lower than 0.5 dBi, as shown in Figure 16a.

(4) $$G=\frac{\mathrm{DQ}}{Q_{r}}$$

G is the antenna gain, D is the antenna radiation direction coefficient, and $Q_{r}$ is radiation loss [33].

(5) $$Q_{r}=\frac{3\lambda_{0}\varepsilon_{r}}{8h}$$

where $\lambda_{0}$ is resonant frequency wavelength, a is the length of the patch, $\varepsilon_{r}$ is dielectric constant of the dielectric plate and h is dielectric plate thickness [34].

(6) $$D=2x^{2}{\left[xsi\left(x\right)+cosx-2+\frac{sinx}{x}\right]}^{-1}$$

(7) $$x=2\pi a\lambda_{0}$$

## 4. Measured and Simulated Results

**Figure 18:**
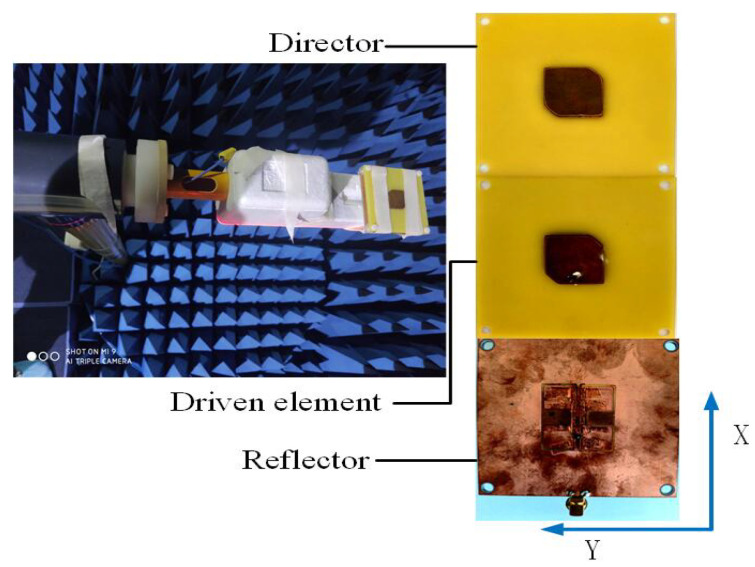
Experimental setup in an anechoic chamber and SUY antenna fabricated prototype.

**Figure 19:**
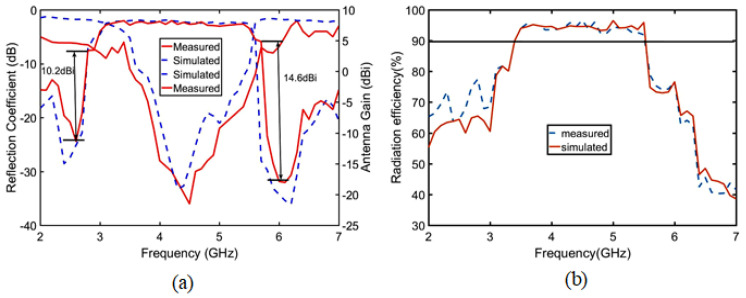
Simulated and measured (**a**) reflection coefficient, gain, and (**b**) efficiency of the SUY antenna.

**Figure 20:**
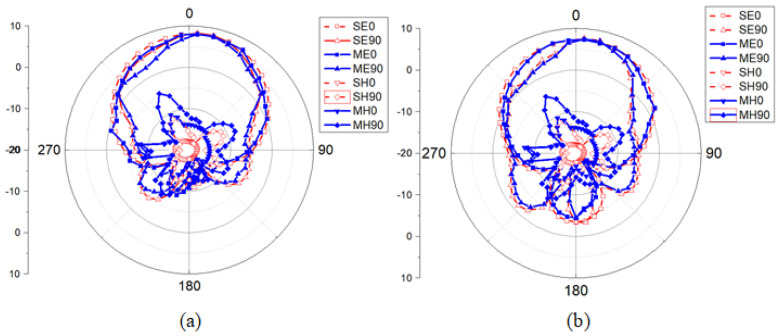
Co-polarization and cross-polarization radiation of (**a**) 4 GHz and (**b**) 5 GHz. SE0 and SE90 are polarized simulations; SH0 and SH90 are cross-polarized simulations; ME0 and ME90 are co-polarized measurements; MH0 and MH90 are cross-polarized measurements.

To verify the validity of our design concept, a prototype of the proposed SUY antenna is fabricated and measured. The experimental setup in the anechoic chamber and the SUY antenna fabricated prototype are shown in Figure 18. The port of the antenna is welded to a 50 ohm SMA. HFSS software is used to simulate the SUY antenna. An R&S ZNB20 vector network analyzer is used to measure the reflection coefficient of the antenna, for which the insertion loss is less than 0.05 dB. Figure 19a shows the comparison between the measured results of the gain and reflection coefficients and the simulation results. The maximum measured filtering suppression reaches 14.6 dBi. The flat gain is measured at 0.6 dBi. Figure 19b and Figure 20 show the simulated and measured values of efficiency and radiation patterns of the SUY antenna. The data were measured in an antenna anechoic chamber, where the signal generator is an R&S microwave signal generator. The output frequency range of this signal generator is from 100 kHz to 20 GHz, and the maximum output power is +11 dBm from 50 MHz to 20 GHz. A step of 1 cm is used to test the radiation and efficiency of the SUY antenna, for which a test distance of approximately 3–5 wavelengths is best. With the adjustment of the step size, the measurement efficiency of the antenna at 3.5–5.5 GHz is more than 90%, as shown in Figure 19b. The radiation angle of the SUY antenna is measured by π/24 step rotations. Figure 20 shows the co-polarization and cross-polarization of 4 GHz and 5 GHz. In manufacturing process of the NZIM structure, welding increases the impedance value, so the copper tape with a thickness of 0.001 mm is used for winding binding. In the test is a certain tilt angle caused by a certain cross-planning error. In the measurement, the small discrepancy may be a result of the manual installation and the error of the measurement system itself.

## 5. Conclusions

In this paper, stereoscopic ultra-wideband (UWB) Yagi–Uda (SUY) antenna with stable gain by NZIM has been discussed and analyzed in detail. Its advantages are high gain, wide bandwidth, and stable gain. The disadvantage is that the three-dimensional size is larger. The combination of coaxial feed and ME structure using electromagnetic superposition and the impedance matching principle achieve high gain and excellent broadband performance. The NZIM with two radiation nulls as well as flat in-band radiation gain is realized in a poof-of-concept prototype of SUY antenna. In order to further improve the stable gain, adjusting the director and driven element impedance enhance the gain of the suppression boundary. The demonstration antenna has been designed and implemented, and the simulated and measured results with good accordance have been presented.

## Figures and Tables

**Table 1:** Performance Comparison with the Literature.

Reference	$\lambda_{0}$ × $\lambda_{0}$ × h (mm^2^)	BW(GHz) & △BW	G & △G (dBi)	△G/△BW (dBi/GHz)
[24]	0.5 × 0.63 ${}^{\left(p\right)}$	5.0–7.5 & 3.5	3.5–5.5 & 2	-
[25]	0.64 × 0.64 ${}^{\left(p\right)}$	0.8–3.0 & 2.2	4–5.2 & 1.2	-
[26]	0.48 × 0.69 × 1 ${}^{\left(s\right)}$	1.8–2.8 & 1	6.5–9 & 2.5	2.5
[20]	1.2 × 1.1 ${}^{\left(p\right)}$	8.5–9.5 & 1	5.0–8.3 & 3.3	2
[27]	0.9 × 1.1 ${}^{\left(p\right)}$	8.5–10 & 1.5	5.5–7 & 1.5	1
This work	0.7 × 0.7 × 0.05 ${}^{\left(s\right)}$	3.5–5.5 & 2.0	7.7–8.7 & 1.0	0.5

(*p*) is plane structure and (*s*) is the stereoscopic structure.

**Table 2:** Dimensions of the proposed SUY antenna.

Parameter	Value (mm)	Parameter	Value (mm)
L1	60	L2	32
L3	11	L4	23
W4	23	H5	3
W1	13	p1	5
p2	5	R1	2

## Data Availability

Not applicable.
